# Deprescribing in Real Time: Hospitalized Septuagenarian With Polypharmacy

**DOI:** 10.7759/cureus.40699

**Published:** 2023-06-20

**Authors:** Tolulope Famuyiro, Alexia Montas, Taylor Tanoos, Trisha E Obinyan, Mukaila Raji

**Affiliations:** 1 Department of Geriatrics, Baton Rouge General Medical Center, Baton Rouge, USA; 2 Department of Family and Community Medicine, Baton Rouge General Medical Center, Baton Rouge, USA; 3 Department of Nursing, Baton Rouge General Medical Center, Baton Rouge, USA; 4 Department of Pharmacy, Baton Rouge General Medical Center, Baton Rouge, USA; 5 Department of Internal Medicine-Division of Geriatrics & Palliative Medicine, University of Texas Medical Branch, Galveston, USA; 6 Department of Preventive Medicine and Population Health, University of Texas Medical Branch, Galveston, USA

**Keywords:** pill burden, polypharmacy, deprescribing, older adults, inappropriate prescribing

## Abstract

Polypharmacy is a common and potentially preventable contributor to recurring emergency room visits, hospitalization, morbidity, and mortality. Its consequences are magnified in older adults due to the age-related decrease in functional and physiologic reserves, increased blood-brain barrier permeability, and altered drug metabolism, among others. In this article, we describe a case of polypharmacy in a septuagenarian to highlight the deprescribing approach implemented by the inpatient care team and to offer patient-centered insights to clinicians (primary care providers and hospitalists) when making deprescribing decisions. The overarching aim of this article is to build on existing literature regarding polypharmacy, prescribing cascades, and deprescribing in the context of what matters most and aligns with patient health priorities. This article highlights the importance of good geriatric medication reconciliation stewardship to avoid harm.

## Introduction

Polypharmacy is often defined as the use of five or more medications. It is a common and potentially preventable contributor to recurring emergency room visits, frequent hospital admissions, early development or progression of geriatric syndromes, morbidity, and mortality [1-3]. It accounts for about 10% of emergency room visits and hospital admissions [1,2]. Polypharmacy is prevalent among frail, community-dwelling older adults [1-3]. The etiology of polypharmacy is multifactorial. It includes patient and provider level factors such as healthcare providers' limited experience managing the complexity of geriatric care, clinicians' perceived fear of undertreating patients when deprescribing, patients’ lack of medication oversight due to poor social support, and attachment to medications without evidence-based clinical benefits [2,3]. As increasing age is linked with multi-morbidity, the need to optimize treatment using current evidence-based guidelines becomes imperative. Efforts to optimize the treatment of chronic conditions among vulnerable older adults are constantly challenged by a downstream detrimental outcome-increased frailty, morbidity, and mortality. In the US, more than a third of older adults are taking more medications than is medically necessary or are receiving new medication(s) for the treatment of the side effect created by another medication (prescribing cascade) [2]. In this instance, the intention to uphold the principle of beneficence is negated when the risk from treatment outweighs the benefit [1,2]. 

## Case presentation

A community-dwelling, 75-year-old African American female presented to our hospital with progressive functional decline, acute mentation change, and presumed new onset seizure. Seizure was witnessed and detailed by her octogenarian husband. The patient’s husband noticed sudden jerking body movement with upward eye-rolling, followed by transient mutism. She slumped over and slid off the bed. This episode reportedly lasted a few minutes; it resolved before the emergency response team arrived at the scene. The emergency response team found the patient on the floor by her bedside. The patient was awake but too confused to obtain reliable history. The patient’s husband alluded that, in the preceding month, she had been unusually sleepier. He attributed her “less conversational” demeanor to ongoing chemotherapy treatment with bortezomib (Velcade) for multiple myeloma; hence, he did not feel the need to mention the change in demeanor as a concern to their primary care provider (PCP). Of note, her last PCP appointment was two months prior to the seizure episode. She had no known prior history of seizure and denied head strike, fever, chills, headache, vomiting, palpitation, or focal deficit. 

Her past medical history included non-insulin dependent type 2 diabetes (hemoglobin A1c: 5.2%), hyperlipidemia, morbid obesity (body mass index 39), heart failure with preserved ejection fracture, essential hypertension, anxiety, depression, chronic low back pain, left adenosquamous lung cancer (pT3N2) status post-lobectomy/chemotherapy/radiation on immunotherapy, multiple myeloma (on bortezomib and Prednisone), insomnia, chronic hypoxic respiratory failure on 2 liters of oxygen via nasal cannula, chronic bronchitis, GERD, functional paraparesis due to frailty and deconditioning (Clinical Frailty Scale 6), and chronic kidney disease stage 3a. Premorbid baseline, she was dependent on her husband for all activities of daily living (ADLs) except feeding, grooming, and toileting. She required one-person assistance for transfer and used a walker and wheelchair for ambulation. Despite her functional limitations, the patient's wish was to receive care in her home (she refused any post-acute care recommendations). Her husband is the primary and sole caregiver. Although her husband denied caregiver fatigue, he expressed that his wife would benefit from more home health visit time (currently, she gets home healthcare visits three times weekly). Her husband could not provide an accurate mental list of all her medications, but he agreed to retrieve her medication bag from home. 

The patient was brought to the emergency room by emergency medical service. En route to the hospital, no repeat seizure event was witnessed. The patient had a Glasgow coma score of 11 (eye-opening 4, best motor response 6, best verbal response 3), elevated blood pressure (169/60 mm Hg), normal heart rate, respiratory rate, and oxygen saturation 94% on 2L of oxygen via nasal cannula. She was awake, but intermittently confused, mostly responding with nods to “Yes'' and “No” questions. Physical examinations showed no evidence of painful distress or meningeal irritation. Cardiac, lung, and abdominal examination was unremarkable, except for 1+ (mild) pedal edema and stage 1 sacral pressure injury. Gait was not assessed.

Laboratory tests revealed normocytic anemia, acute kidney injury with superimposed chronic kidney disease stage 3a, and hypoalbuminemia. See Table 1 for laboratory and reference values.

**Table 1:** Pertinent laboratory values Blood cells per microliter (109/L), gram per deciliter (g/dL), milligram per deciliter (mg/dL), milliliters of cleansed body per minute per body surface area (mL/min/1.73 m2), micromoles per liter (μmol/L), millimeters per hour (mm/hr), femtoliter (fL) MCR: Mean corpuscular volume, ESR: erythrocyte sedimentation rate; BUN: blood urea nitrogen

Hematology			
Laboratory test	Result	Reference interval	Units
WBC (Total White Blood Cell)	9.56	4.5 - 11	10^9^/L
Hb (Hemoglobin)	9.6	12 - 16	g/dL
Platelet	242	15 - 400	10^9^/L
MCV	88	80 - 99	fL
ESR	2	1-20	mm/hr
Biochemistry			
Laboratory test	Result	Reference interval	Units
Sodium	142	134 -144	mmol/L
Potassium	3.8	3.5 - 5.2	mmol/L
Glucose	78	65 - 99	mg/dL
BUN	18	6 - 24	mg/dL
Creatinine	1.2	0.6 - 1.1	mg/dL
eGFR (Estimated Glomerular Filtration Rate)	54	90 - 120	mL/min/1.73 m^2^
Calcium	8.4	8.7 – 10.2	mg/dL
Albumin	1.9	3.4 – 5.4	g/dL
Magnesium	1.8	1.8 -2.6	mg/dL

Sedimentation rate, alcohol, and salicylic acid levels were at undetectable levels. The urine drug screen was positive for opiates, benzodiazepine, and cannabinoids. Urinalysis and blood culture were negative. A chest X-ray noted stable left upper lung zone pulmonary opacity comparable to her prior X-ray. An electrocardiogram revealed sinus rhythm with normal QTc (399). Head computerized tomography revealed multifocal parenchymal calcifications (chronic) but negative for stroke (10/10 ASPECT score). Brain magnetic resonance imaging was also unremarkable. 

The team was cognizant of the fact that taking bortezomib could cause dizziness and fatigue but would not explain the mentation change. Since infectious, neurologic, and metabolic workups were significantly unremarkable, and the team could not ascertain this to be a true seizure, the care team agreed to defer initiating anti-epileptic therapy while focusing on streamlining her medications. To determine the chronology of recently added and discontinued medications, the clinical pharmacist accessed the patient’s outpatient medication record. This enabled the pharmacist to identify profound medication discrepancies: Her home medication bag had 12 more medications that were not present on her outpatient pharmacy medication record. Outpatient medication consisted of 16 medications (excluding Velcade (bortezomib) from nine drug classes (12 oral pills, three inhalers, and one nasal spray). Her home medication bag had 28 medications from 16 drug classes (24 pills, three inhalers, and one nasal spray). The discrepancies between the outpatient pharmacy’s list and the patient’s home medication list were the presence of duplicate benzodiazepine, multiple psychotropics, opioids, expired bottle of furosemide, and several over-the-counter supplements (e.g., multivitamins, tetrahydrocannabinoid (THC) gummies, vitamin B12). See Table 2 for the medication list.

**Table 2:** Consideration for adjustment and discontinuation based on the patient’s home medication and pharmacy’s dispensary medication list gram (g); miligram (mg); microgram (mcg); GERD: gastroesophageal reflux disease

Indication/Diagnosis	Patient's pharmacy dispensary list	Patient’s home medication list (reviewed at bedside)	Potentials for Reduction	Considerations for Continuation, Reduction or Discontinuation	Hospital discharge medication recommendations
Chronic bronchitis	Advair inhaler	Advair inhaler	No	Medically indicated	Continue Advair inhaler
Chronic bronchitis	Albuterol inhaler	Albuterol inhaler	No	Medically indicated	Continue Albuterol inhaler
Chronic bronchitis	Umeclidinium inhaler	Umeclidinium inhaler	No	Medically indicated	Continue Umeclidinium inhaler.
Hypertension	Carvedilol 25 mg twice daily	Carvedilol 25 mg twice daily	No	Medically indicated	Continue Carvedilol 25 mg twice daily.
Hypertension	Amlodipine 5 mg daily	Amlodipine 5 mg daily	No	Medically indicated	Continue Amlodipine 5 mg daily
Vitamin D deficiency and osteopenia	Cholecalciferol 2000 unit daily	Cholecalciferol 2000 unit daily	No	Medically indicated.	Continue Cholecalciferol 2000 units daily.
Multiple myeloma	Prednisone 20 mg daily x 7 days, then 10 mg daily x 7 days, 5 mg daily x 7 days	Prednisone 20 mg daily x 7 days, then 10 mg daily x 7 days, 5 mg daily x 7 days	No	Prescribed by an oncologist. Currently on tapered dose.	Continue Prednisone 20 mg daily x 7 days, then 10 mg daily x 7 days, 5 mg daily x 7 days
Anxiety/depression	Duloxetine 60 mg twice daily	Duloxetine 60 mg twice daily	No	Medically indicated	Continue Duloxetine 60 mg twice daily.
Depression, anorexia, insomnia	Mirtazapine 15 mg nightly	Mirtazapine 15 mg nightly	No	Medically indicated	Continue Mirtazapine 15mg nightly.
Allergic rhinitis	Fluticasone 50 mcg actuation spray	Fluticasone 50 mcg actuation spray	No	Continue use as needed	Continue Fluticasone 50 mcg actuation spray as needed.
Hyperlipidemia	Pravastatin 20 mg daily	Pravastatin 20 mg daily	No	Indicated for hyperlipidemia. Time to benefit ~ 12-24 months. Limited benefit from use when life expectancy is shorter than lag time.	Continue Pravastatin 20 mg daily.
Chronic low back pain	Acetaminophen 650 mg every 6 hours as needed for pain.	Tylenol 650 mg every 6 hours as needed for pain.	No	Not to exceed 4 g total daily use. Cautious use with other acetaminophen containing combination opioids	Continue Acetaminophen 650 mg every 6 hours as needed for pain.
Multiple myeloma treatment	Bortezomib (Velcade)	Bortezomib (Velcade)	No	To be continued by her oncologist.	Continue Bortezomib (Velcade)
Chronic low back pain	Oxycodone-Acetaminophen 10-325 mg 1 tablet three times daily	Oxycodone-Acetaminophen 10-325 mg 1 tablet three times daily	Yes	Dose adjusted to as needed. Do not exceed the total dose of 4 g when used concomitantly with Tylenol.	Oxycodone-Acetaminophen 10-325 mg changed/reduced to 1 tablet three times daily as needed for pain.
Insomnia		Lorazepam (Ativan) 0.25 mg twice daily as needed for sleep.	Yes	Beers criteria PIM Unclear if she was taking both or alternating between temazepam and lorazepam.	To prevent withdrawal symptoms, lorazepam (Ativan) 0.25 mg reduced to daily as needed for sleep.
GERD		Omeprazole 40 mg daily	Yes	Consideration for gastrointestinal prophylaxis related to steroid use. Dose reduced. Can be discontinued if no longer on steroids.	Omeprazole dose reduced to20 mg daily
Insomnia and nausea		Tetrahydrocannabinoid (THC) gummies	Yes	Complementary medication/supplement. Mixed report on benefit for insomnia	THC (cannabis gummies)- continued per patient’s request.
Primary stroke prevention	Aspirin 81 mg daily	Aspirin 81 mg daily	Yes	Aspirin discontinued. Limited benefit for primary stroke prevention	
Allergic rhinitis	Cetirizine 10 mg daily as needed.	Cetirizine 10 mg daily as needed.	Yes	Cetirizine discontinued due to sedating effects. Evidence favors intranasal steroid as first line for allergic rhinitis.	
Hypertension	Irbesartan 150 mg daily	Irbesartan 150 mg daily	Yes	Irbesartan discontinued due acute kidney injury and blood pressure at goal with amlodipine and carvedilol	
Anxiety		Venlafaxine 75 mg twice daily	Yes	Venlafaxin discontinued on pharmacy dispensary med list. Combined use of Venlafaxine with another SSRI, worsens seizure and hypertension.	
Multiple myeloma		Dexamethasone 4 mg once a day, every other day	Yes	Dexamethasone discontinued. Replaced with prednisone by oncologist.	
Insomnia, anxiety		Temazepam 15 mg nightly	Yes	Temazepam discontinued. Temazepam is a short acting benzodiazepine with higher withdrawal risk. Unclear if she was taking both or alternating between these two meds.	
Nausea		Compazine 5 mg every 6 hours as needed for nausea.	Yes	Compazine discontinued on pharmacy dispensary report. Can induce seizure.	
Anxiety/depression		Buspirone 7.5 mg twice daily	Yes	Buspirone discontinued on pharmacy dispensary report. Buspirone can cause or worsen confusion	
Over-the-counter supplement		Multivitamin daily	Yes	Multivitamin discontinued. Limited benefit to multivitamin use.	
Vitamin B12 (Cyanocobalamin) deficiency		Vitamin B12 supplement daily	Yes	Vitamin B12 discontinued since Vitamin B12 level was supratherapeutic.	
Dependent edema and history of heart failure		Furosemide 20 mg daily	Yes	Furosemide discontinued on pharmacy dispensary report.	

The patient and spouse agreed with the team to simplify her medications, including discarding expired or duplicate medications. The clinical pharmacist cross checked for drug-drug interaction. We used deprescribing guides (e.g., MedStopper and Beer's criteria potentially inappropriate medication (PIM)) to identify which medications to taper or discontinue to prevent withdrawal symptoms. Her medications were streamlined to eight oral medications, two as-needed medications, three inhalers, one nasal spray, and one over-the-counter medication (THC gummies; requested to be retained by the patient). See Table 2 for details on medication reduction.

Her hospital course was complicated by a brief episode of psychosis (auditory hallucination without disruptive behavior), likely related to the changes made to her medication or delirium-triggered hospitalization. With close monitoring, her symptoms regressed. By day 3 of admission, she was more coherent, sitting up in bed, but still requiring one-person assist to transfer from bed to chair. Physical therapy recommended post-acute care, but the patient expressed her preference for being discharged to home with home health. The discharge coordinator arranged 48-hours post-discharge follow-up with her primary care physician. The patient’s caregiver was encouraged to use a pill box to organize medications at home. On the day of discharge, the physician and discharge nurse met with the patient and caregiver to review her medications using the teach-back technique. She has been doing well since discharge, and there has been no report of repeat seizure. 

## Discussion

“Polypharmacy” is loosely defined as the use of many medications, but “many” may be interpreted as the use of five or more medications, the presence of high-risk or Beer’s criteria PIMs, and concurrent use of multiple medications for the treatment of a single condition, among others. While a unified definition may not currently exist, a systematic review article by Masnoon et al. raised important points about standardizing the term “polypharmacy” to eliminate confusion among healthcare providers and the public. Specifically, the article highlighted the need to reappraise the definition of polypharmacy by focusing less on the numeric count and more on appropriate prescribing based on best clinical practice utilization [3]. 

Appropriate prescribing in this context would involve weighing several factors simultaneously, such as the time-to-benefit against life expectancy while considering the patient’s other co-morbidities and care preferences, deprescribing medication with no evidence-based benefit, redosing medication with changing kidney function, and eliminating indefinite use of medications that fail to achieve intended therapeutic objectives or when indication for use no longer exists. Perhaps, embracing the term “medication inappropriateness or inappropriate prescribing” over “polypharmacy” would assure consistency and uniformity [4-15]. 

The most misprescribed medications among older adults include psychotropics, anticholinergics, and analgesics. Other less reported but often overutilized medications include Vitamin B-12 (cobalamin) supplements, gabapentinoids, proton pump inhibitors (PPIs), and bisphosphonate [9-13]. According to the American Academy of Family Physicians, vitamin B12 supplements should only be prescribed indefinitely to bariatric surgery patients, while short-term use (up to six months) is recommended for chronic metformin users, treatment of macrocytic anemia, peripheral neuropathy, or cognitive impairment with confirmed cobalamin deficiency. Periodic monitoring of vitamin B12 levels should be included in the care plan to inform the prescriber’s decision to extend the duration of treatment beyond the recommended timeline. An article by Bongiovanni et al. identified prolonged use (more than 12 weeks after surgery) of gabapentinoids among post-operative older adults despite increasing risk of delirium and death [10]. Overutilization of PPIs and bisphosphonate among frail, debilitated older adults contributes to the pill burden conundrum [11-13]. 

The healthcare provider factors that contribute to inappropriate prescribing include prescribers’ “perceived” fear of litigation from patient undertreatment when deprescribing, “perceived” fear of incurring patient dissatisfaction, time constraints in implementing pill burden taper in a busy outpatient practice, limited physician oversight on mid-level providers automating medication refills, and limited healthcare providers' experience managing the complexity of geriatric care with patients’ preference and priorities [4]. When providers refuse to honor patients' requests for medications, they know that they risk reprisals in the form of lower ratings. The fear of incurring patient dissatisfaction is a disincentive for deprescribing medications with high dependence risks (e.g., benzodiazepine). This steers providers to the “patient pleasing” mode. Automated medication refills- an unprecedented gain from telemedicine to address social isolation and limited access to care created by the COVID-19 pandemic-are now threatened by the increase in automated medication refill requests [5-9]. 

The patient factors that contribute to inappropriate prescribing include attachment to medications with limited evidence-based clinical benefit, low health literacy, and inadequate medication oversight by caregivers. Elderly spousal caregivers whose health problems compete with those of their partner are at risk of caregiver burnout [5-9, 14,15]. Low health literacy affects a person’s ability to understand, appraise, and participate in shared decision-making related to their personal health. It is linked to treatment non-compliance or reluctance to discontinue medications with questionable efficacy (e.g., dietary supplements like yohimbe for weight loss or chondroitin sulfate for osteoarthritis). Table 3 summarizes the considerations for appropriate and inappropriate prescribing.

**Table 3:** Summary of key considerations for appropriate and inappropriate prescribing

Key considerations for appropriate prescribing
The time-to-benefit against life expectancy
Patient’s co-morbidities (especially co-existing dementia and frailty status) and care preferences
Deprescribing medication with no evidence-based benefit
Redosing medication with changing kidney function
Eliminating indefinite use of medications that fail to achieve intended therapeutic objectives
Eliminating indefinite use of medications when indication for use no longer exists.
Contributors to inappropriate prescribing
Patient's trust in over-the-counter medications and dietary supplements
Prescribers’ hesitation to broach the topic or negotiate deprescribing with patients
Fear of litigation with “perceived” undertreatment when deprescribing
Time constraints in implementing pill burden taper in a busy practice
Clinicians struggle with other competing demands- administrative duties
Limited oversight on automated medication refills
Treating a drug side effect with a new drug - “prescribing cascade”
Limited experience managing the multi-complexity of geriatric care
Limited organizational resources to engage interdisciplinary team (e.g clinical pharmacist, geriatrician, etc. ) in care planning /treatment
Juxtaposing the reality of implementing deprescribing intervention to align with the patients’ preference and priorities.
Fear of unfavorable patient satisfactory rating of healthcare providers if medications were stopped—especially benzodiazepine and opioids.
The “patient pleasing” syndrome

It remains unclear how and why this profound polypharmacy was missed. Plausible explanations for failing to identify a significant medication-related issue include the presence of an elderly-spousal caregiver, limited social support to the patient and caregiver, lack of recognition of polypharmacy side effects due to limited health literacy, fragmented care resulting from seeing multiple healthcare providers, and communication gaps between the primary care provider, home health staff, and other involved specialists.

Clinicians, patients, and caregivers play an integral role in perpetuating inappropriate prescribing and advocating deprescribing [4,16,17]. The ongoing effort to find a sustainable strategy to curb inappropriate prescribing led to the development of the Team Approach to Polypharmacy Evaluation and Reduction (TAPER) model by Mangin et al. TAPER is a three-step approach that can be implemented in both acute and post-acute/ambulatory care settings. It uses a secure platform to gather the patient’s medication and medical history, screen for medication inappropriateness, and flag at-risk electronic medical records (EMRs) [18]. The flagged chart is transmitted to the clinical pharmacist, who reviews medication dispensary, assesses for drug-drug interaction meets with the patient and caregiver/family to inquire about potential side effects, and identifies medications suitable for drug holiday, reduction, or discontinuation. The pharmacist’s recommendation is then disseminated to the physician for implementation. TAPER can be integrated into inpatient and outpatient settings. It confers a huge opportunity to intercept fragmented patient care and irrational prescribing or medication misuse. For TAPER to be sustainable, insurance companies and healthcare organizations must be willing to reimburse the time and effort of those championing this project at their various institutions. 

Older adults with frailty, dementia, and other chronic multi-morbidities or those on psychotherapeutic and high-risk medications need routine evaluation by a geriatrician and/or a geriatric-psychiatrist. Multi-dose blister packs (in place of traditional pill bottles) should be offered to patients when cognitive impairment is identified as a barrier to treatment compliance. Patients and caregivers should be encouraged to participate in brown bag review i.e., to bring all home medications, including over-the-counter medications and dietary supplements, to their doctors’ appointments. Medication at risk for injudicious prescribing e.g., bisphosphonate, PPIs, vitamin B12, gabapentinoids, etc. should be flagged on the EMRs and scrutinized before refills are initiated. If a patient is reluctant about deprescribing a certain medication, proposing a stepwise approach with contingencies might be appropriate. For patients with multiple medical conditions, prescribing medications with multiple therapeutic benefits to reduce pill burden is encouraged. As employed in this case, duloxetine was continued (instead of venlafaxine) to treat depression and pain, while mirtazapine was continued for treatment of depression, poor appetite, and insomnia. Deprescribing tools (e.g., MedStopper and Screening Tool of Older Person’s Prescriptions (STOPP)/Screening Tool to Alert Doctors to Right Treatment (START)) can improve safe prescribing in older adults, and the teach-back technique and closed-loop communication also help to ascertain patients/ caregivers’ understanding [15-17]. To improve patient safety during transition of care from the acute to post-acute care setting, the use of synchronous bi-directional (physician-to-physician telephone call) communication is encouraged. If this is not feasible, asynchronized unidirectional communication (sending a detailed discharge summary to the patient’s primary care provider) could suffice [19]. 

## Conclusions

Deprescribing can be challenging yet rewarding for both the patient and the clinician. To address inappropriate medication use in older adults, clinicians can implement strategies such as engaging interdisciplinary teams, promoting closed-loop communication between care providers, exploring patients’ health priorities, and matching patients’ care needs with the safest, most effective, and least cumbersome treatment. Every opportunity to deprescribe favors treatment compliance, reduces dependence on high-risk medications (e.g., opioids, psychotropics, and tranquilizing medications), and averts accidental overdose on duplicate or expired medication. 
